# Validation of an electrochemical sensor based on gold nanoparticles as a point-of-care test for quantitative determination of glycated hemoglobin

**DOI:** 10.1371/journal.pone.0276949

**Published:** 2023-06-29

**Authors:** Kanyarat Boonprasert, Thipaporn Tharavanij, Chiravoot Pechyen, Khanittha Ponsanti, Benchamaporn Tangnorawich, Vithoon Viyanant, Kesara Na-Bangchang

**Affiliations:** 1 Graduate Program in Bioclinical Sciences, Chulabhorn International College of Medicine, Thammasat University, Pathum Thani, Thailand; 2 Department of Medicine, Faculty of Medicine, Thammasat University, Pathum Thani, Thailand; 3 Department of Materials and Textile Technology, Faculty of Science and Technology, Thammasat University, Pathum Thani, Thailand; 4 Department of Physics, Faculty of Science and Technology, Thammasat University, Pathum Thani, Thailand; Hamadan University of Medical Sciences, ISLAMIC REPUBLIC OF IRAN

## Abstract

Monitoring the level of glycated hemoglobin (HbA1c) has become the gold standard measure for diabetes mellitus (DM) diagnosis and control, used in conjunction with fasting blood glucose (FBG) and oral glucose tolerance test. This study aimed to investigate the applicability of a newly developed nanoparticle-based electrochemical sensor—multiwalled nanotubes incorporated with gold nanoparticles (POCT-HbA1c^MWCNTs/AuNPs^)—used as a routine point-of-care test (POCT) for detection of HbA1c for the diagnosis of DM. Finger-prick and venous blood samples were collected from 108 DM and 98 non-DM subjects to determine HbA1c and total hemoglobin by POCT-HbA1c^MWCNTs/AuNPs^ compared with the standard HPLC method. The performance of the POCT-HbA1c^MWCNTs/AuNPs^ was evaluated using the standard cut-off HbA1c level of >6.5%. The test’s sensitivity, specificity, positive predictive value, and negative predictive value were 100.00%, 90.32%, 87.23%, and 100.00%, respectively. The probability of DM diagnosis in a subject with HbA1c >6.5% (positive predictive value) was 87.23% (82/94). The accuracy of the POCT-HbA1c^MWCNTs/AuNPs^ was 94.18%, with a %DMV (deviation from the mean value) of 0.25%. The results indicate satisfactory assay performance and applicability of the POCT-HbA1c^MWCNTs/AuNPs^ for diagnosis of DM using the cut-off criteria of HbA1c >6.5.

## Introduction

Diabetes mellitus (DM) is a chronic metabolic disorder categorized by elevated levels of plasma glucose due to defects in insulin secretion (type I), insulin action (type II), or both. Several metabolic diseases can follow, which may lead to permanent damage, including amputation, retinopathy, neuropathy, and cardiovascular diseases [1]. The growing prevalence of DM is a global concern. Approximately 541 million people are at increased risk of developing type 2 DM. About half of those with DM are unaware of their state of illness, which may lead to delay in proper diagnosis and management [2]. While there is no definitive remedy for DM, disease progression and complications could be minimized by rapid diagnosis and close monitoring of blood glucose levels [3]. However, fasting blood glucose (FBG) concentrations only provide a snapshot of blood glucose at the time of blood sample collection and do not provide a long-term view of disease management [4]. In addition, FBG level is affected by many different factors and is not the most effective biomarker for DM diagnosis and control [5]. Monitoring glycated hemoglobin (HbA1c) level has become the gold standard measure for DM diagnosis and control, used in conjunction with FBG and oral glucose tolerance test [6]. HbA1c is hemoglobin A bound with glucose; it is a common glycosylated protein found in the body formed by the non-enzymatic covalent bonding of glucose to the *N*-terminal valine of the beta-globin chain [7]. Its concentration depends on the erythrocyte lifespan and the blood glucose level. The term HbA1c also refers to the ratio of HbA1c to total hemoglobin (Hb) concentration, and the %normal range of HbA1c is 5% to 20% of total Hb. The cut-off value of HbA1c of ≥6.5% is recommended for the diagnosis of DM, according to the American Diabetes Association and World Health Organization guidelines [8]. Pre-DM is defined as having an HbA1c of 5.7–6.4%, and non-DM is defined as having an HbA1c of 4.0–5.6% [9, 10]. Furthermore, HbA1c is also a useful biomarker of long-term glycemia and a good predictor of lipid profile. Monitoring glycemic control could, thus, benefit DM patients at risk of cardiovascular disease [11]. Accurate and precise methods for HbA1c measurement are therefore required for better glycemic control.

Currently, various methods are available for measuring HbA1c levels in the blood [12]. These methods are based on separation according to ionic charge (ion-exchange chromatography, electrophoresis) [13, 14], affinity binding (affinity chromatography for HbA1c) [15], enzymatic assays [16], colorimetric assays [17], or immunoassays [18, 19]. HPLC is considered the gold standard for HbA1c analysis due to its accuracy in simultaneous quantifying HbA1c and Hb concentrations in whole blood, used to obtain the HbA1c percentage [20, 21]. Nevertheless, these methods have limitations such as complexity, availability, the requirement for experienced personnel, cost, time consumption, and interference from endogenous and exogenous compounds [22, 23]. Point-of-care testing (POCT) of HbA1c is a user-friendly test that can more quickly and accurately diagnose DM than the standard method. Several sensor-based POCTs for quantitatively determining HbA1c concentration in blood have been developed. These sensors for HbA1c detection offer potentially superior analytical and device design with regard to test sensitivity, cost, simplicity, robustness, and miniaturization [24, 25]. Among the various sensors that have been developed, the electrochemical type can respond to biological samples rapidly and is suitable for miniaturization, rapid on-site detection, portability, and flexibility [26].

In the present study, the applicability of the newly developed nanoparticle-based electrochemical sensor—multiwalled nanotubes corporated with gold nanoparticles (POCT-HbA1c^MWCNTs/AuNPs^) as a routine POCT for detection of HbA1c for diagnostic purposes was investigated [27]. The gold nanoparticles (AuNPs) were prepared using green chemistry instead of conventional chemistry, which uses toxic chemicals. This green chemistry approach is cost-effective, convenient, and environmentally friendly. The obtained materials were deposited onto the surface of MWCNTs, and their electrochemical responses were measured using cyclic voltammetry (CV). These modified electrodes were then used for HbA1c detection in DM patients with high sensitivity. The performance of this test was validated in blood samples obtained from confirmed DM patients and non-DM subjects.

## Materials and methods

### Electrochemical sensor

**Fig 1:**
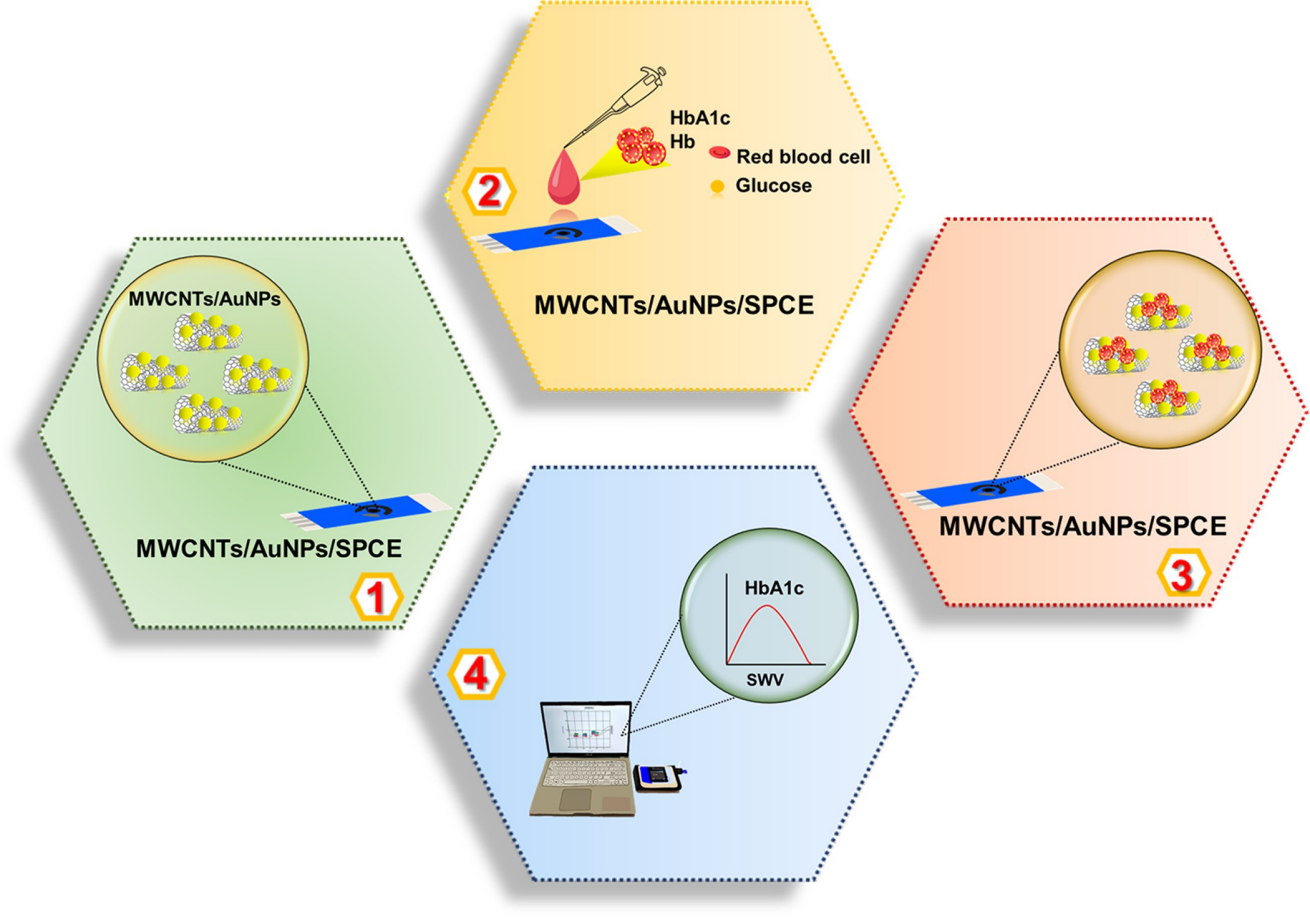
The format of POCT-HbA1c ^MWCNTs/AuNPs^ (adapted from ref. [27]).

The development of this electrochemical sensor based on multiwalled carbon nanotubes/gold-nanoparticles (MWCNTs/AuNPs) using a modified screen-printed carbon electrode (SPCE) for measurement of HbA1c and total Hb in blood samples has previously been described in detail [27]. In brief, a sensor based on gold nanoparticles (AuNPs) was synthesized from passion fruit (*Passiflora edulis*) peel extracts. The prepared AuNPs (2 mL) were mixed with multiwalled carbon nanotubes (MWCNTs, 5 mg) in various ratios (1:1, 1:2, and 2:1) to form MWCNTs/AuNPs composites. The prepared MWCNTs/AuNPs were characterized for morphology (transmission electron microscope: TEM), particle size (dynamic light scattering), crystallinity (X-ray diffraction), functional groups (Fourier transform infrared spectra), UV-absorbance (UV-vis-spectrophotometry), and elemental composition (energy-dispersive X-ray spectrometry). The nanoparticles are spherical with a diameter of approximately 18 nm, a face-centered cubic crystal structure, and a maximum UV-vis peak at 550 nm. The small AuNPs and the MWCNTs’ intermeshing improve the composite material’s conductivity. This property, in conjunction with the carbon nanotubes, results in good stability and good support for the fabrication of the electrochemical sensing system. The MWCNTs/AuNPs composite was drop-casted on the SPCE for working electrode preparation. The working electrode (MWCNTs/AuNPs/SPCE) was air-dried to remove all solvents. The electrochemical response of modified electrodes to MWCNTs/AuNPs (1:1, 1:2, 2:1) was measured using cyclic voltammetry (CV) by PalmSens4 Potentiostat/Galvanostat/Impedance Analyzer (PalmSens, Netherlands). The electrochemical cells consisted of a MWCNTs/AuNPs/SPCE electrode (working electrode), Ag/AgCl electrode (reference electrode), and a platinum electrode (counter electrode). The format of POCT-HbA1c^MWCNTs/AuNPs^ is shown in Fig 1.

The responses of MWCNTs/AuNPs/SPCE electrodes to different concentrations of total Hb and HbA1c were investigated by square wave voltammetry (SWV) over the concentration ranges of 5–13 g/dL and 0.186–2.044 g/dL, respectively, to construct the calibration curves. The selectivity of MWCNTs/AuNPs/SPCE electrodes for HbA1c and total Hb to other interfering molecules were also evaluated under identical conditions. The interfering substances were 3 mM glucose, 3 mM galactose, 3 mM fructose, 0.20 mM uric acid (UA), 0.05 mM ascorbic acid (AA), and 0.5 wt% bovine serum albumin (BSA). Concentrations of the interfering substances were chosen based on their average level in the human body [28, 29]. All were 5-fold diluted with phosphate buffer for the consistency of whole blood sample preparation for HbA1c analysis.

### Measurement of HbA1c and total Hb concentrations in blood samples

The applicability of the POCT-HbA1c^MWCNTs/AuNPs^ was validated in 206 human blood samples, in comparison with the standard HPLC method. Twenty microliters (10 μL) of finger-prick blood were mixed with 50 μL of hemolyzing reagent (0.9% tetradecyl ammonium bromide 26.5 mmol/L), and an aliquot of cell suspension (5 μL) were dropped onto an MWCNTs/AuNPs electrode. The electrochemical response of HbA1c and Hb were measured using SWV by PalmSens4 Potentiostat/Galvanostat/Impedance Analyzer (PalmSens, Netherlands). The percentage of HbA1c was calculated according to DCCT/NGSP: HbA1c (%) = (HbA1c/Hb)*91.5+2.15 [30, 31].

### Patients

The study was approved by the Ethics Committee, Faculty of Medicine, Thammasat University (approval number 157/2564, project number MTU-EC-00-4-062/64). The study was conducted at Thammasat Chalermprakiet Hospital and the Thammasat University Center of Excellence in Pharmacology and Molecular Biology of Malaria and Cholangiocarcinoma, following the guidelines outlined in the Declaration of Helsinki. Written informed consent was obtained from all subjects before study participation. A minimum of 138 study subjects (69 each for DM and non-DM subjects) were recruited for the study. The required sample size was estimated using the formula:

$$n=\frac{\left[{\left(Z_{\frac{\propto }{2}}\right)}^{2}\times (1-P)\right]}{d^{2}}$$

Where n = sample size, Z = 95% confidence interval (1.96), and d (%error) = 5%. P is the sensitivity (0.96) and specificity (0.90) of the POCTs reported in previous studies [32].

Inclusion criteria for the DM group were (i) clinical- and laboratory-confirmed DM of all types and stages, (ii) males or females (non-pregnant and non-lactating), (iii) aged between 20 and 70 years, and (iv) willing to participate in the study. Exclusion criteria were those with (i) significant abnormalities found during physical examination, (ii) significant diseases that may affect study results, (iii) abnormal coagulation or concurrent use of anticoagulant/antiplatelet agents, and (vi) participation in any other study within the past 90 days.

Inclusion criteria for the non-DM group were (i) males or females (non-pregnant and non-lactating), (ii) aged between 20 and 70 years, (iii) those with body mass index (BMI) between 18 and 25 kg/m^2^, (iv) non-smokers and non-alcoholic drinkers, and (vii) willing to participate in the study. Exclusion criteria were those with (i) significant abnormalities found during physical examination, (ii) having or have had hepatitis B or hepatitis C or HIV infection, (v) abnormal coagulation or concurrent use of anticoagulant/antiplatelet agents, and (vi) participation in any other clinical study within the past 90 days.

Blood samples were collected from all volunteers after overnight fasting (8–10 hours). Venous blood samples (3 mL each) were collected in EDTA tubes and transferred to the Bangkok Pathology-Laboratory Co. Ltd. for determination of HbA1c (Automated HbA1c, BioRad D-10 HPLC), total Hb, red blood cells (RBC) count, hematocrit, mean cell volume (MCV), mean cell hemoglobin (MCH), mean cell hemoglobin concentration (MCHC), red blood cell distribution width (RDW), platelet count, and white blood cells (WBC) count. The cut-off value of DM diagnosis was set at HbA1c ≥6.5%.

### Evaluation of the performance of POCT-HbA1c^MWCNTs/AuNPs^

#### POCT-HbA1c^MWCNTs/AuNPs^ performance

The performance (sensitivity, specificity, positive and negative predictive values, and accuracy) of the POCT-HbA1c^MWCNTs/AuNPs^ was evaluated by comparing the measured HbA1c values with those measured by the reference laboratory method using the standard cut-off HbA1c level of ≥6.5% [9]. The test performances were estimated as follows:

Sensitivity (%) = TP/(TP+FN)

Specificity (%) = TN/(TN+FP)

Positive predictive value (%) = TP/(TP+FP)

Negative predictive value (%) = TN/(TN+FN)

Accuracy (%) = (TP+TN)/(TP+TN+FP+FN)

False-positive (%) = Number of misdiagnosed DM cases by POCT/Total number of negative cases by reference method

False-negative (%) = Number of misdiagnosed non-DM cases by POCT/Total number of positive cases by reference method

Where TP = True positive, TN = True negative, FP = False positive, FN = False negative.

#### Test agreement analysis

The 95% confidence interval and the mean relative error were estimated by Bland-Altman plot and PasinggeBablok regression analysis (MedCalc Software, Mariakerke, Belgium) to determine the consistency of the HbA1c values measured by POCT-HbA1c^MWCNTs/AuNPs^ and those measured by the reference laboratory method.

### Statistical analysis

Statistical analysis was performed using SPSS 17.0 software. Quantitative variables are summarized as median (range) and mean (SD) for non-normal and normal variables, respectively. Deviation from the mean for HbA1c values was measured by the POCT test, based on values measured by the reference laboratory method, and are expressed as %DMV (% deviation from mean value). The correlation between the concentrations of the two quantitative variables was determined using Spearman’s or Pearson’s correlation test for variables with non-normal or normal distribution, respectively. Statistical significance was set at α = 0.05.

## Results

### Electrochemical detection of HbA1c and total Hb

**Fig 2:**
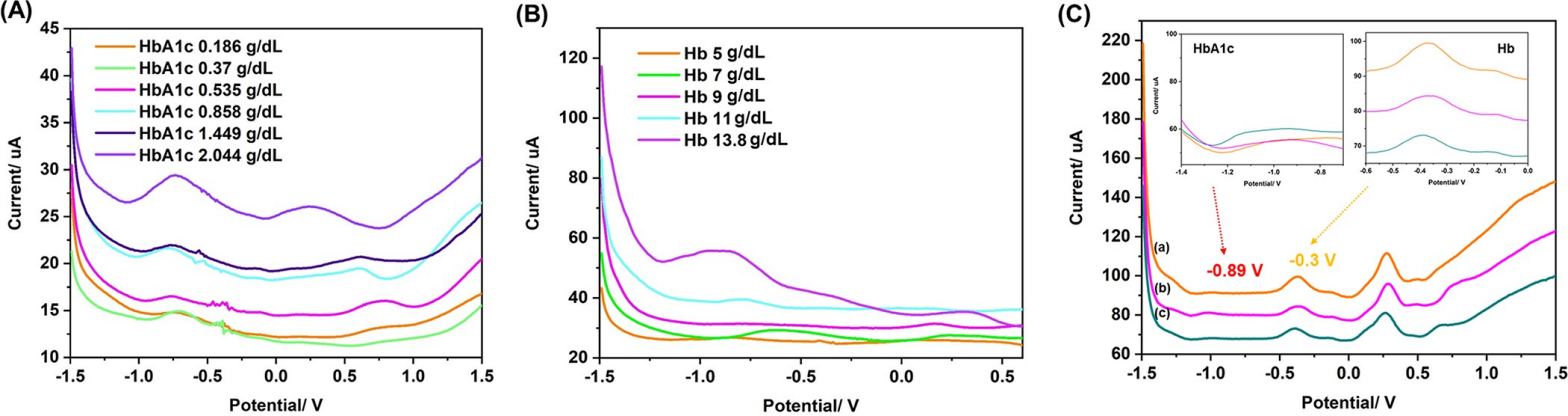
The responses of MWCNTs/AuNPs electrodes (SWV) to various concentrations of HbA1c and total Hb. (A) SWV voltammograms for the detection of HbA1c, (B) SWV voltammograms for the detection of total Hb, and (C) SWV voltammograms for the detection of HbA1c and total Hb in blood samples: (a) human blood sample No. 1, (b) human blood sample No. 2, and (c) human blood sample No. 3 (adapted from ref. [27]).

The responses of MWCNTs/AuNPs electrodes (SWV) to various concentrations of HbA1c and total Hb are shown in Fig 2A and 2B. HbA1c and total Hb showed oxidation potential peaks at -0.89 V and -0.3 V, respectively. The intensity of the peak current was proportional to the concentrations of HbA1c and total Hb in the concentration ranges of 0.186–2.044 g/dl and 5–13.8 g/dl, respectively. The position of the oxidation peaks remained unchanged with varying concentrations of HbA1c and total Hb, which confirms their selective response to these electrodes. The electrical current and Hb1Ac concentration relationship was linear: I_P_ (μA) = 8.6488C + 11.122, r = +0.9645). The limit of detection (LOD) of HbA1c was 0.01 g/dL (99% confidence level). For total Hb, the equation for linear regression was: I_P_ (μA) = 1.228C + 21.287 (r = +0.9004). The LOD was 0.01 g/dL (99% confidence level).

**Fig 3:**
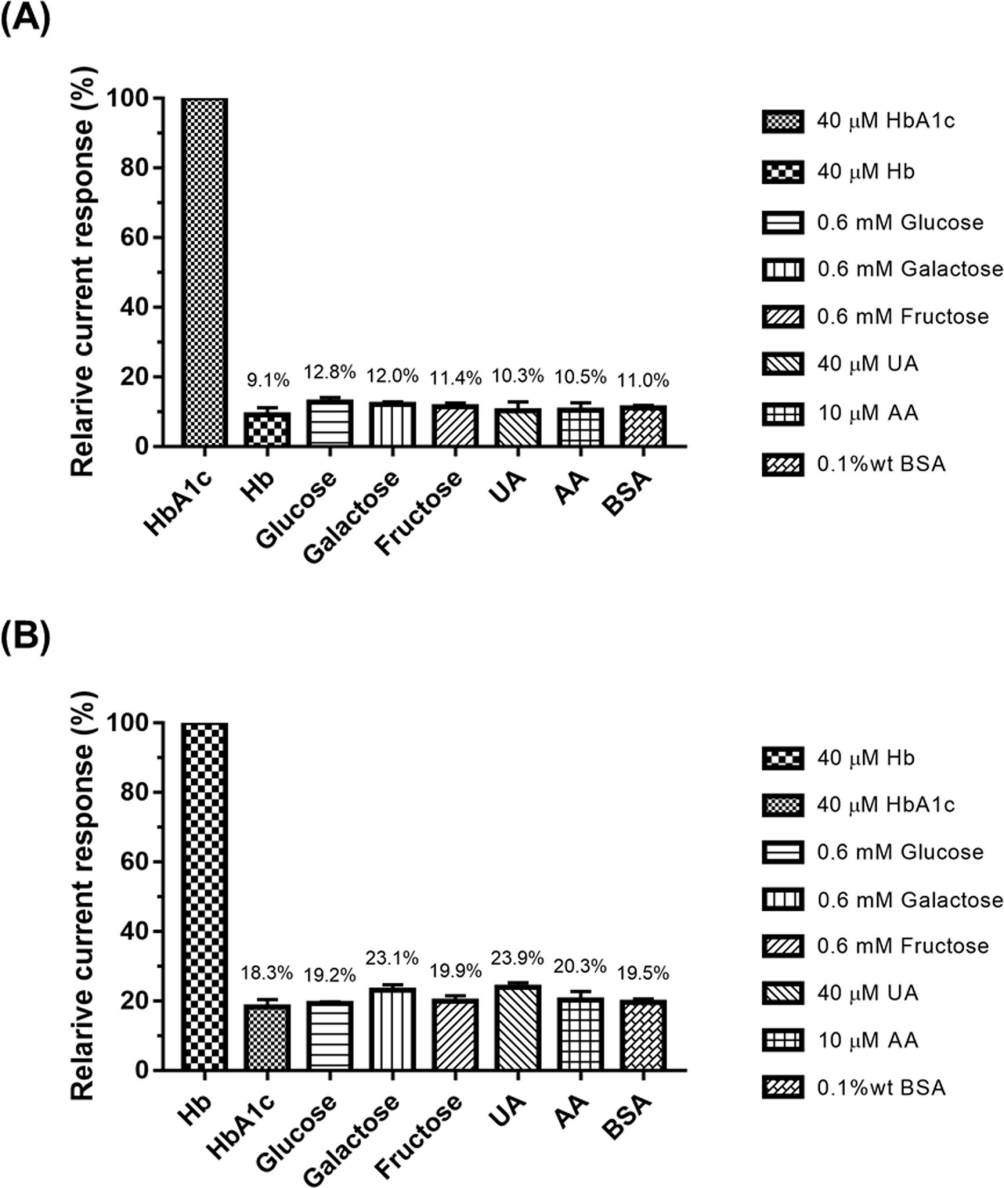
The selectivity test of MWCNTs/AuNPs electrodes. Effects of interfering substances on (A) HbA1c detection, and (B) total Hb detection.

Selectivity of the developed MWCNTs/AuNPs electrodes was evaluated against small molecules, *i*.*e*., glucose, galactose, and fructose, and large molecules, *i*.*e*., BSA and Hb under identical experimental conditions. As shown in Fig 3A, it was clear that small molecules did not interfere with HbA1c detection. A similar observation was made with the large molecules—Hb and BSA. The results indicate negligible interfering effects by endogenous substances for HbA1c detection by MWCNTs/AuNPs electrodes. It was noted however for the relatively low effects of some interfering substances on total Hb (Fig 3B). Therefore, these interfering substances at high concentrations may cause a false measurement of total Hb by MWCNTs/AuNPs electrodes.

### Analysis of the performance of POCT-HbA1c^MWCNTs/AuNPs^

Performance analysis of the POCT-HbA1c compared with the reference laboratory method was performed in 108 DM and 98 non-DM subjects. The median (range) HbA1c concentrations measured by POCT-HbA1c^MWCNTs/AuNPs^ in DM and non-DM patients were 0.64 (0.36–0.93) and 0.48 (0.22–0.64) g/dL, respectively [corresponding to 7.19 (5.18–11.89) and 6.03 (4.10–6.40)% of total Hb, respectively]. The corresponding HbA1c concentrations measured by the reference method were 0.70 (0.39–1.78) and 0.45 (0.25–0.66) g/dL, respectively [corresponding to 7.10 (4.80–14.00) and 5.30 (4.30–6.40)% of total Hb, respectively]. Median (range) total Hb concentrations measured by POCT-HbA1c^MWCNTs/AuNPs^ in DM and non-DM patients were 12.65 (10.26–14.71) and 11.46 (9.65–12.58) g/dL, respectively. The corresponding total Hb concentrations measured by the reference method were 13.10 (10.50–15.50) and 13.00 (8.80–15.20) g/dL, respectively.

#### POCT-HbA1c^MWCNTs/AuNPs^ performance

To evaluate the clinical applicability of the POCT-HbA1c^MWCNTs/AuNPs^, the HbA1c values measured by the POCT-HbA1c^MWCNTs/AuNPs^ (S1 Table) were compared with that measured by the reference laboratory method (S1 Table) using the cut-off value of 6.5% (Table 1). The sensitivity and specificity of the POCT-HbA1c were 100.00% (82/82) and 90.32% (112/124), respectively. The probability of correct DM diagnosis in a subject with HbA1c ≥6.5 (positive predictive value) was 87.23% (82/94). The likelihood of a truly negative test result in a subject with HbA1c <6.5 (negative predictive value) was 100.00% (112/112). The accuracy of the POCT-HbA1c^MWCNTs/AuNPs^ expressed as a proportion of true positives and true negatives (correctly classified by POCT-HbA1c^MWCNTs/AuNPs^) in all subjects was 94.18%, with a %DMV of 0.25%.

**Table 1:** Analysis of HbA1c levels using POCT-HbA1c^MWCNTs/AuNPs^ based on a 6.5% cut-off value.

HbA1c measured by POCT (%)	HbA1c measured by reference laboratory method (%)	
	HbA1c ≥ 6.5	HbA1c < 6.
HbA1c ≥ 6.5	82	12
HbA1c < 6.5	0	112
**Total**	82	124

Data are presented as the number of subjects (total number = 206).

#### Test agreement analysis

**Fig 4:**
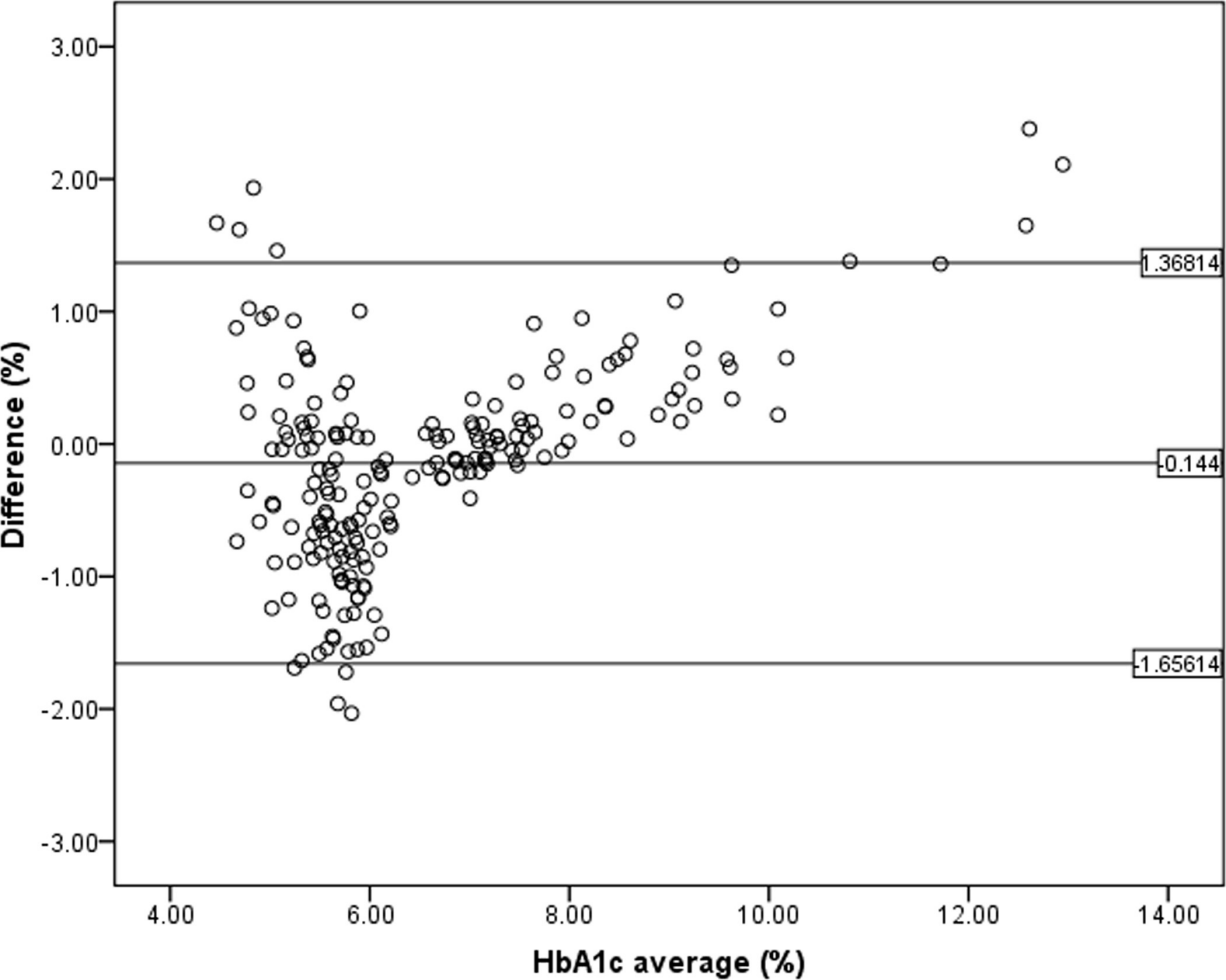
Bland-Altman plot analysis for accuracy of HbA1c measured by POCT-HbA1c compared with the reference method.

Bland-Altman plot analysis of the HbA1c analyzed by POCT-HbA1c^MWCNTs/AuNPs^ and the reference method was performed, and results showed that only 12 subjects had values outside the 95% CI (confidence interval) of the range of agreement limit (-1.65614 to 1.36814), suggesting that 94.18% of the values measured by the POCT-HbA1c^MWCNTs/AuNPs^ were in agreement with those measured by the reference method (Fig 4).

#### Clinical application of POCT-HbA1c^MWCNTs/AuNPs^

**Fig 5:**
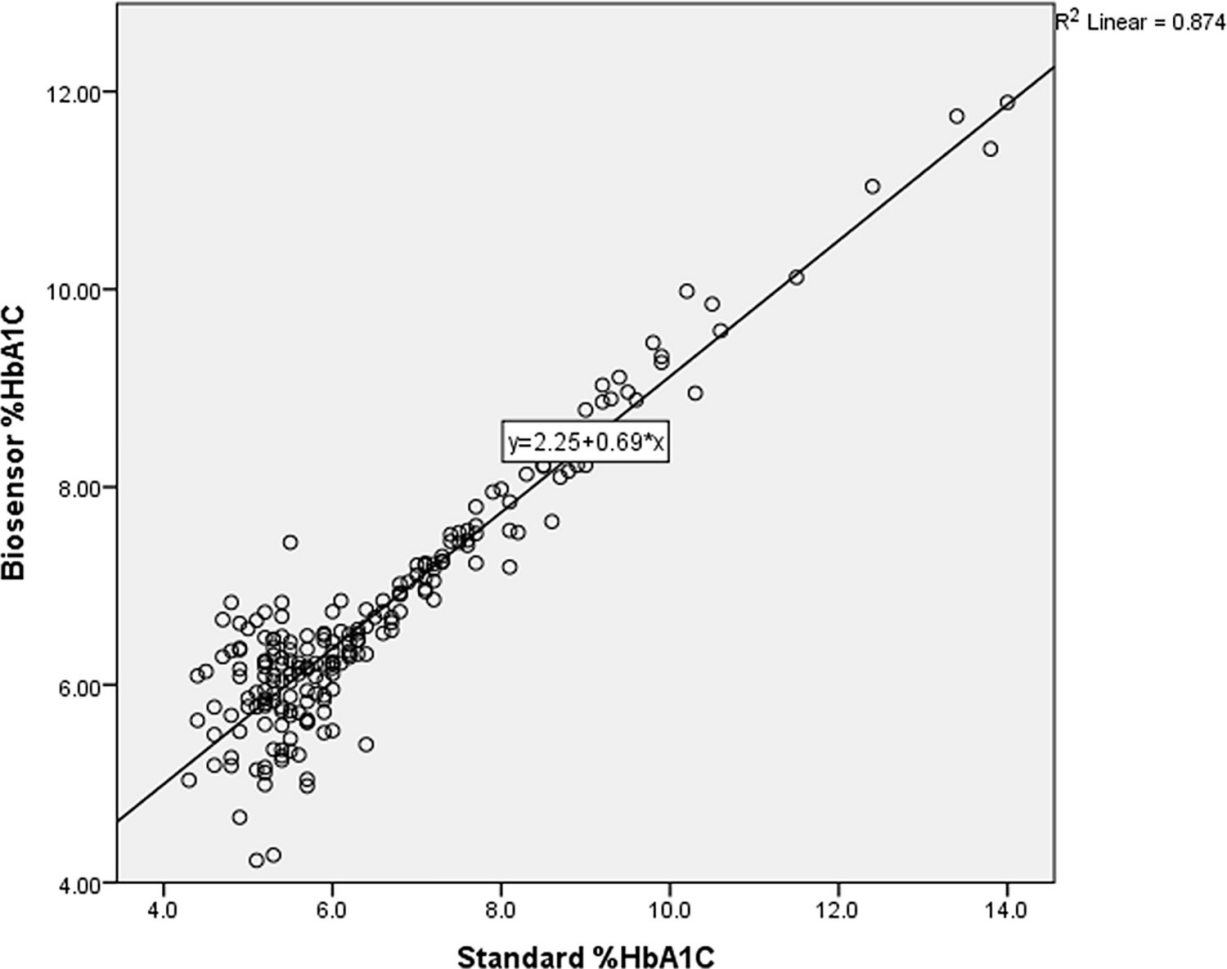
Correlation between HbA1C concentrations measured by the POCT-HbA1c ^MWCNTs/AuNPs^ and the reference laboratory method.

Clinical application of the POCT-HbA1c^MWCNTs/AuNPs^ was evaluated in 108 DM and 98 non-DM subjects. The demographic and laboratory data of the DM and non-DM subjects are summarized in Table 2. Excellent linear correlation (*p*<0.0001, Y = 2.25+0.69*X, r = +0.874) was found between the HbA1c values measured by the POCT-HbA1c and the reference laboratory method (Fig 5). There was a moderate linear correlation between FBG and HbA1c levels measured by the standard method in DM and non-DM subjects (r = +0.552, *p*<0.0001).

**Table 2:** Demographic, glucose level, and hematological data of DM and non-DM subjects.

Parameter		DM	Non-DM
**Gender (n, %)**	Male (n, %)	37 (33.90%)	14 (14.03%)
	Female (n, %)	71 (65.10%)	84 (85.70%)
**Age (year)**	Mean (SD)	59.8 (12.7)	32.6 (9.1)
	Median (range)	60.9 (20.5–86.3)	31.0 (15.9–57.8)
**Body weight (kg)**	Mean (SD)	70.5 (15.7)	59.0 (11.8)
	Median (range)	68.0 (37.3–130.0)	56.0 (38.5–88.0)
**BMI**	Mean (SD)	27.13 (5.28)	22.76 (4.19)
	Median (range)	25.95 (16.56–48.93)	21.67 (15.13–34.38)
**Glucose (mg/dL)**	Mean (SD)	179.3 (79.8)	108.0 (22.2)
	Median (range)	156.0 (68.0–442.0)	101.0 (79.0–222.0)
**Hemoglobin (g/dL)**	Mean (SD)	13.0 (1.2)	12.9 (1.2)
	Median (range)	13.1 (10.5–15.5)	13.0 (9.8–15.2)
**Hematocrit (%)**	Mean (SD)	40.0 (3.8)	40.2 (3.9)
	Median (range)	39.9 (31.6–48.5)	40.0 (32.2–48.8)
**Platelet (x10** ^**3**^ **/μL)**	Mean (SD)	264.80 (71.18)	290.92 (59.30)
	Median (range)	2604.00 (69.00–507.00)	282.00 (152.00–450.00)
**RBC (x10** ^**6**^ **/μL)**	Mean (SD)	4.69 (0.59)	4.77 (0.59)
	Median (range)	4.58 (3.76–6.84)	4.73 (3.78–6.77)
**HbA1c (g/dL)**	Mean (SD)	0.77 (0.27)	0.45 (0.08)
	Median (range)	0.70 (0.39–1.78)	0.45 (0.25–0.66)
**HbA1c (%)**	Mean (SD)	7.57 (1.80)	5.33 (0.42)
	Median (range)	7.10 (4.80–14.00)	5.30 (4.30–6.40)
**MCV (fL)**	Mean (SD)	85.94 (7.87)	85.01 (9.07)
	Median (range)	87.30 (60.4–98.9)	87.90 (63.00–97.10)
**MCH (pg)**	Mean (SD)	28.60 (2.80)	27.34 (3.22)
	Median (range)	28.60 (20.10–32.60)	27.90 (19.40–32.40)
**MCHC (g/dL)**	Mean (SD)	32.80 (0.87)	32.12 (1.03)
	Median (range)	32.80 (30.10–35.10)	32.20 (27.90–33.60)
**RDW (%)**	Mean (SD)	12.93 (1.02)	13.06 (1.35)
	Median (range)	12.70 (11.00–16.60)	12.80 (11.00–18.00)
**WBC (x10** ^**3**^ **/μL)**	Mean (SD)	7.43 (2.04)	6.52 (1.48)
	Median (range)	7.36 (3.76–15.76)	6.55 (2.49–10.58)
**Neutrophil %)**	Mean (SD)	59.65 (8.56)	54.31 (9.12)
	Median (range)	59.10 (34.70–41.80)	54.50 (23.80–73.30)
**Lymphocyte (%)**	Mean (SD)	32.55 (8.17)	38.18 (8.68)
	Median (range)	32.60 (17.10–56.70)	36.50 (16.60–68.70)
**Monocyte (%)**	Mean (SD)	3.60 (1.71)	2.85 (1.09)
	Median (range)	2.90 (1.00–9.00)	2.50 (1.00–6.10)
**Eosinophil (%)**	Mean (SD)	3.56 (2.64)	3.96 (1.92)
	Median (range)	2.90 (0.50–19.70)	3.60 (1.00–10.00)
**Basophil (%)**	Mean (SD)	0.57 (0.29)	0.71 (0.35)
	Median (range)	0.50 (0.0–1.6)	0.60 (0–2)

Data are presented as number (%) or mean (SD) and median (range) values.

Correlation analysis of the HbA1c values measured by the POCT-HbA1c^MWCNTs/AuNPs^ and hematological parameters (Hb, Hct, MCV, MCHC, and RDW) was performed to determine hematological factors that could influence the measurement of HbA1c by POCT-HbA1c^MWCNTs/AuNPs^. Only two parameters were found to be significantly correlated with the measured HbA1c by POCT-HbA1c^MWCNTs/AuNPs^. Total Hb concentration (measured by POCT-HbA1c^MWCNTs/AuNPs^ and reference method) was weakly (r = +0.310) but significantly (*p*<0.0001) correlated with HbA1c measured by POCT-HbA1c^MWCNTs/AuNPs^. On the other hand, a negative correlation (r = -0.214, *p* = 0.002) was found between MCHC and HbA1c values measured by POCT-HbA1c^MWCNTs/AuNPs^.

## Discussion

Glycosylated Hb is formed *via* a spontaneous non-enzymatic reaction between glucose and the *N*-terminal residues of the beta-globin chain in Hb, generating unstable Schiff base intermediates. Then, the intermediate undergoes irreversible Amadori rearrangement to synthesize the stable ketoamine [33]. The accomplishment of quantitative HbA1c testing provides in-depth and accurate detection for clinical application. A number of HbA1c POCT devices are currently available in clinical settings which use either affinity-based separation or immunoassay detection methods. An electrochemical detection sensor has also been developed as a POCT for HbA1c. Each device type has some limitations, such as test complications requiring multi-step sample preparation [34] or the requirement of large blood sample volume [35]. Furthermore, most devices are subject to interferences from Hb variants (HbC, HbE, HbS, and others) and modified Hb (carbamylated and acetylated Hb, or labile HbA1c) [36]. The analysis showed that most of the HbA1c POCT devices have a mean negativity bias compared to laboratory assays and a high variability among the bias values within device types. Recently, a gold nanoflower-modified electrochemical sensor based on 4-mercaptophenylboronic acid was developed to quantitatively determine HbA1c [37]. In this study, we investigated the clinical applicability of the POCT-HbA1c^MWCNTs/AuNPs^ previously developed in our laboratory [27] for the accurate diagnosis of DM. The test was sensitive and specific for the determination of HbA1c in the blood (from both venipuncture and finger-prick), exploiting the catalytic property of HbA1c to reduce H_2_O_2_ to produce an electrochemical signal. The selectivity of total Hb and HbA1c relies on the signal of SWV. Hb is measured using an electrochemical sensing method in real-time through successive oxidization and deoxidization processes (a redox signal) of Fe^2+^/ Fe^3+^ in heme protein. HbA1c is glycosylated hemoglobin formed when the amino terminus of the beta-chain of Hb. Due to the difference in chemical structures of HbA1c and Hb, they produce different oxidation or redox peak current [38]. The selectivity of total Hb and HbA1c depends on the square wave voltammetry (SWV) signal. The peak current of the sensor in differential pulse voltammogram can be distinguished among total Hb and HbA1c. As shown in Fig 2, total Hb and HbA1c present an oxidation potential peak respectively at -0.3 V and -0.89 V, which confirms the selective response to these electrodes for the detection of total Hb and HbA1c. Apart from the difference in oxidation potentials, we also confirmed the identity of both proteins using reference standards. The results were in agreement with the previous report [39].

The sensor was prepared from gold nanoparticles (AuNPs) in multiwalled carbon nanotubes (MWCNTs) as MWCNTs/AuNPs composites. The apparent advantage of this test is the use of green synthesis (green chemistry) for synthesising AuNPs from passion fruit peel instead of toxic chemicals. In addition, it is a label-free method that assures rapid detection. AuNPs were electrochemically deposited onto the SPCE. The starch composition in the passion fruit peel with different amylose pectins acts as a reducing agent in the electrochemical reaction [40]. This label-free method could assure rapid detection with a volume as small as 20 μl of blood. Only a single-step pre-treatment of the blood samples is required to remove plasma interference, followed by lysis of the RBC. The POCT-HbA1c^MWCNTs/AuNPs^ provide determination of both HbA1c and total Hb concentrations in a single reaction. This feature not only reduces testing time but also reduces errors from the use of different reactions.

To demonstrate the clinical applicability of the developed POCT-HbA1c^MWCNTs/AuNPs^ electrochemical sensor, the concentrations of HbA1c in blood samples from DM and non-DM subjects measured by the POCT-HbA1c^MWCNTs/AuNPs^ and the reference laboratory methods were compared. Results show high sensitivity (100%), specificity (90.32%), accuracy (94.18%), and excellent application prospects in clinical practice. The probability of a diagnosis of DM in subjects with HbA1c level ≥6.5% (positive predictive value) was 87.23%, while the probability of misdiagnosis of DM in subjects with HbA1c level <6.5% (negative predictive value) was 100.00%. The agreement of the values measured by both methods was excellent (r = +0.874). The Bland-Altman plot suggested that 94.18% of the values measured by POCT-HbA1c^MWCNTs/AuNPs^ were in agreement with those measured by the standard reference method. HbA1c levels were correlated with FBG, as previously reported [11]. The measurement of HbA1c as a percentage of total Hb relies on the measurement accuracy of both HbA1c and total Hb concentrations. A weak linear correlation was found between total Hb concentration (measured by standard and POCT-HbA1c^MWCNTs/AuNPs^ methods) and HbA1c concentration measured by POCT-HbA1c^MWCNTs/AuNPs^ (r = +0.32). This suggests that total Hb has little influence on the HbA1c concentration as measured by POCT-HbA1c^MWCNTs/AuNPs^. The POCT-HbA1c^MWCNTs/AuNPs^ could have broad clinical applicability in patients regardless of the Hb concentration. The influence of total Hb concentration on HbA1c determination has been reported in patients with hemoglobinopathies such as thalassemia. The hematocrit varies among individuals, which leads to differences in total Hb. The low hematocrit level could be due to the following reasons: the amount of RBC, as well as the amount of Hb in RBCs, are not enough to completely fill the surface of the sensor, and/or HbA1c does not reflect the actual ratio. On the other hand, the MCHC value, which relates to the volume of RBC, affected the HbAc1 level measured by POCT-HbA1c^MWCNTs/AuNPs^ in a reverse direction; the higher the MCHC concentration, the lower the HbA1c concentration measured by POCT-HbA1c^MWCNTs/AuNPs^ (r = -0.214, *p* = 0.002). Falsely high HbA1c levels have been reported to be associated with conditions that prolong the lifespan of RBCs (*e*.*g*., iron deficiency anemia), severe hypertriglyceridemia (>1,750 mg/dL), severe albuminemia (>20 mg/dL), alcohol intake, and administration of some drugs (*e*.*g*., salicylate, opioids) [41]. On the other hand, falsely low HbA1c levels have been reported to be associated with conditions that shorten the lifespan of RBCs, splenomegaly, pregnancy, and administration of some drugs (*e*.*g*., ribavirin, cephalosporin, and levofloxacin).

In conclusion, the results of this study indicate satisfactory assay performance and applicability of the POCT-HbA1c^MWCNTs/AuNPs^ for the diagnosis of DM using the standard cut-off criteria of an HbA1c of ≥6.5%. The POCT-HbA1c^MWCNTs/AuNPs^ is an accurate and easy-to-use tool that provides test results on the spot and has become an effective tool in establishing DM diagnosis, especially in vulnerable or hard-to-reach populations. It does not require fasting before measurement, and the sample can be stored at 4 C° for at least two weeks for cases in which measurement cannot be performed immediately. A user-friendly format that employs a smartphone application (MyA1c) is being developed for clinical use in routine diagnosis of DM. The implication of using POCT-HbA1c^MWCNTs/AuNPs^ in medical treatment decision-making to care for DM patients and control patient outcomes needs to be further evaluated.

## Supporting information

S1 Table HbA1c levels using POCT-HbA1c^MWCNTs/AuNPs^ and reference laboratory method. (DOCX)
