# Supplementary material for: Validation of an electrochemical sensor based on gold nanoparticles as a point-of-care test for quantitative determination of glycated hemoglobin
Source: PLoS One. 2023 Jun 29;18(6):e0276949. doi: 10.1371/journal.pone.0276949 (PMC10309628; doi:10.1371/journal.pone.0276949)
Supplement: S1 Table — (DOCX) [file pone.0276949.s001.docx]

**S1 Table. HbA1c levels using POCT-HbA1c ^MWCNTs/AuNPs^ and reference laboratory method.**

| **Sample No.** | **HbA1c (%)** | | **Difference** | **Mean** |
| --- | --- | --- | --- | --- |
|  | **Standard method (HPLC)** | **POCT** |  |  |
| 1 | 5.40 | 4.45 | 0.95 | 4.93 |
| 2 | 4.90 | 6.16 | -1.26 | 5.53 |
| 3 | 4.80 | 5.26 | -0.46 | 5.03 |
| 4 | 5.50 | 5.33 | 0.17 | 5.41 |
| 5 | 5.90 | 6.45 | -0.55 | 6.18 |
| 6 | 5.70 | 5.06 | 0.64 | 5.38 |
| 7 | 5.30 | 6.30 | -1.00 | 5.80 |
| 8 | 5.20 | 6.23 | -1.03 | 5.71 |
| 9 | 4.60 | 4.95 | -0.35 | 4.78 |
| 10 | 5.50 | 6.21 | -0.71 | 5.85 |
| 11 | 5.20 | 5.86 | -0.66 | 5.53 |
| 12 | 5.30 | 5.81 | -0.51 | 5.56 |
| 13 | 5.00 | 4.54 | 0.46 | 4.77 |
| 14 | 4.80 | 5.69 | -0.89 | 5.25 |
| 15 | 5.80 | 5.72 | 0.08 | 5.76 |
| 16 | 4.60 | 5.19 | -0.59 | 4.89 |
| 17 | 5.80 | 4.34 | 1.46 | 5.07 |
| 18 | 5.30 | 5.35 | -0.05 | 5.32 |
| 19 | 5.20 | 5.16 | 0.04 | 5.18 |
| 20 | 5.00 | 5.04 | -0.04 | 5.02 |
| 21 | 5.40 | 5.24 | 0.16 | 5.32 |
| 22 | 4.80 | 6.83 | -2.03 | 5.82 |
| 23 | 5.20 | 4.99 | 0.21 | 5.10 |
| 24 | 5.70 | 5.65 | 0.05 | 5.67 |
| 25 | 5.40 | 6.69 | -1.29 | 6.05 |
| 26 | 5.80 | 6.08 | -0.28 | 5.94 |
| 27 | 4.30 | 5.03 | -0.73 | 4.67 |
| 28 | 5.50 | 4.51 | 0.99 | 5.01 |
| 29 | 5.80 | 3.87 | 1.93 | 4.83 |
| 30 | 5.40 | 5.34 | 0.06 | 5.37 |
| 31 | 5.00 | 5.86 | -0.86 | 5.43 |
| 32 | 5.10 | 4.22 | 0.88 | 4.66 |
| 33 | 5.70 | 4.98 | 0.72 | 5.34 |
| 34 | 5.70 | 5.04 | 0.66 | 5.37 |
| 35 | 5.00 | 5.78 | -0.78 | 5.39 |
| 36 | 4.40 | 5.64 | -1.24 | 5.02 |
| 37 | 5.30 | 4.28 | 1.02 | 4.79 |
| 38 | 5.10 | 5.14 | -0.04 | 5.12 |
| 39 | 5.50 | 6.10 | -0.60 | 5.80 |
| 40 | 5.10 | 5.77 | -0.67 | 5.44 |
| 41 | 5.30 | 5.83 | -0.53 | 5.57 |
| 42 | 4.60 | 5.50 | -0.90 | 5.05 |
| 43 | 5.70 | 4.77 | 0.93 | 5.23 |
| 44 | 5.30 | 3.63 | 1.67 | 4.47 |
| 45 | 5.60 | 5.72 | -0.12 | 5.66 |
| 46 | 5.60 | 5.29 | 0.31 | 5.45 |
| 47 | 4.90 | 4.66 | 0.24 | 4.78 |
| 48 | 5.50 | 5.45 | 0.05 | 5.48 |
| 49 | 4.70 | 6.28 | -1.58 | 5.49 |
| 50 | 6.00 | 5.53 | 0.47 | 5.77 |
| 51 | 6.40 | 5.40 | 1.00 | 5.90 |
| 52 | 5.70 | 6.18 | -0.48 | 5.94 |
| 53 | 5.30 | 6.46 | -1.16 | 5.88 |
| 54 | 5.20 | 5.95 | -0.75 | 5.57 |
| 55 | 5.50 | 5.88 | -0.38 | 5.69 |
| 56 | 5.70 | 5.62 | 0.08 | 5.66 |
| 57 | 5.40 | 4.92 | 0.48 | 5.16 |
| 58 | 5.30 | 5.59 | -0.29 | 5.45 |
| 59 | 6.00 | 5.95 | 0.05 | 5.98 |
| 60 | 5.50 | 6.25 | -0.75 | 5.87 |
| 61 | 5.20 | 6.18 | -0.98 | 5.69 |
| 62 | 5.50 | 3.88 | 1.62 | 4.69 |
| 63 | 5.30 | 5.91 | -0.61 | 5.61 |
| 64 | 5.40 | 6.04 | -0.64 | 5.72 |
| 65 | 5.50 | 5.69 | -0.19 | 5.59 |
| 66 | 4.50 | 6.13 | -1.63 | 5.32 |
| 67 | 5.40 | 6.21 | -0.81 | 5.81 |
| 68 | 5.50 | 6.43 | -0.93 | 5.97 |
| 69 | 5.90 | 5.52 | 0.38 | 5.71 |
| 70 | 5.90 | 5.72 | 0.18 | 5.81 |
| 71 | 5.80 | 6.22 | -0.42 | 6.01 |
| 72 | 5.40 | 5.28 | 0.12 | 5.34 |
| 73 | 4.90 | 6.62 | -1.72 | 5.76 |
| 74 | 6.30 | 6.55 | -0.25 | 6.43 |
| 75 | 5.60 | 6.17 | -0.57 | 5.89 |
| 76 | 5.10 | 6.39 | -1.29 | 5.75 |
| 77 | 5.20 | 5.79 | -0.59 | 5.49 |
| 78 | 5.20 | 6.74 | -1.54 | 5.97 |
| 79 | 5.30 | 6.15 | -0.85 | 5.73 |
| 80 | 6.00 | 6.23 | -0.23 | 6.11 |
| 81 | 4.90 | 6.08 | -1.18 | 5.49 |
| 82 | 5.40 | 6.83 | -1.43 | 6.12 |
| 83 | 5.70 | 5.63 | 0.07 | 5.66 |
| 84 | 4.70 | 6.66 | -1.96 | 5.68 |
| 85 | 5.10 | 6.65 | -1.55 | 5.88 |
| 86 | 5.40 | 6.49 | -1.09 | 5.94 |
| 87 | 5.20 | 5.11 | 0.09 | 5.16 |
| 88 | 5.20 | 5.60 | -0.40 | 5.40 |
| 89 | 5.40 | 5.59 | -0.19 | 5.49 |
| 90 | 5.70 | 6.36 | -0.66 | 6.03 |
| 91 | 4.80 | 5.25 | -0.45 | 5.02 |
| 92 | 5.50 | 5.73 | -0.23 | 5.62 |
| 93 | 5.20 | 5.81 | -0.61 | 5.51 |
| 94 | 5.40 | 6.27 | -0.87 | 5.84 |
| 95 | 5.20 | 6.23 | -1.03 | 5.72 |
| 96 | 5.20 | 6.48 | -1.28 | 5.84 |
| 97 | 5.30 | 6.00 | -0.70 | 5.65 |
| 98 | 5.10 | 5.92 | -0.82 | 5.51 |
| 99 | 4.90 | 6.37 | -1.47 | 5.63 |
| 100 | 4.40 | 6.09 | -1.69 | 5.24 |
| 101 | 6.10 | 6.22 | -0.12 | 6.16 |
| 102 | 5.30 | 6.09 | -0.79 | 5.70 |
| 103 | 5.30 | 6.46 | -1.16 | 5.88 |
| 104 | 5.40 | 5.74 | -0.34 | 5.57 |
| 105 | 5.90 | 6.50 | -0.60 | 6.20 |
| 106 | 5.40 | 5.43 | -0.03 | 5.42 |
| 107 | 5.30 | 6.37 | -1.07 | 5.84 |
| 108 | 6.00 | 6.21 | -0.21 | 6.11 |
| 109 | 5.40 | 6.47 | -1.07 | 5.94 |
| 110 | 5.50 | 6.35 | -0.85 | 5.93 |
| 111 | 5.50 | 6.12 | -0.62 | 5.81 |
| 112 | 5.20 | 6.24 | -1.04 | 5.72 |
| 113 | 5.40 | 5.77 | -0.37 | 5.59 |
| 114 | 4.80 | 6.34 | -1.54 | 5.57 |
| 115 | 4.90 | 5.53 | -0.63 | 5.21 |
| 116 | 5.00 | 6.57 | -1.57 | 5.78 |
| 117 | 5.70 | 6.50 | -0.80 | 6.10 |
| 118 | 4.90 | 6.35 | -1.45 | 5.63 |
| 119 | 5.90 | 5.85 | 0.05 | 5.87 |
| 120 | 5.20 | 6.09 | -0.89 | 5.64 |
| 121 | 6.00 | 6.17 | -0.17 | 6.08 |
| 122 | 4.60 | 5.77 | -1.17 | 5.19 |
| 123 | 8.20 | 7.54 | 0.66 | 7.87 |
| 124 | 6.70 | 6.68 | 0.02 | 6.69 |
| 125 | 8.60 | 7.65 | 0.95 | 8.13 |
| 126 | 7.70 | 7.61 | 0.09 | 7.66 |
| 127 | 6.60 | 6.86 | -0.26 | 6.73 |
| 128 | 7.40 | 7.11 | 0.29 | 7.26 |
| 129 | 7.20 | 7.05 | 0.15 | 7.13 |
| 130 | 8.40 | 7.89 | 0.51 | 8.15 |
| 131 | 6.80 | 6.91 | -0.11 | 6.86 |
| 132 | 6.80 | 6.93 | -0.13 | 6.87 |
| 133 | 8.50 | 8.22 | 0.28 | 8.36 |
| 134 | 7.60 | 7.41 | 0.19 | 7.51 |
| 135 | 8.30 | 8.13 | 0.17 | 8.22 |
| 136 | 7.50 | 7.44 | 0.06 | 7.47 |
| 137 | 9.30 | 8.89 | 0.41 | 9.10 |
| 138 | 7.20 | 7.22 | -0.02 | 7.21 |
| 139 | 7.50 | 7.54 | -0.04 | 7.52 |
| 140 | 13.40 | 11.75 | 1.65 | 12.58 |
| 141 | 8.80 | 8.16 | 0.64 | 8.48 |
| 142 | 7.40 | 7.56 | -0.16 | 7.48 |
| 143 | 9.80 | 9.46 | 0.34 | 9.63 |
| 144 | 7.00 | 7.21 | -0.21 | 7.11 |
| 145 | 8.10 | 7.56 | 0.54 | 7.83 |
| 146 | 11.50 | 10.12 | 1.38 | 10.81 |
| 147 | 7.30 | 7.30 | 0.00 | 7.30 |
| 148 | 8.10 | 7.85 | 0.25 | 7.98 |
| 149 | 9.60 | 8.52 | 1.08 | 9.06 |
| 150 | 5.90 | 6.52 | -0.62 | 6.21 |
| 151 | 7.70 | 7.53 | 0.17 | 7.62 |
| 152 | 6.60 | 6.85 | -0.25 | 6.73 |
| 153 | 7.30 | 7.25 | 0.05 | 7.28 |
| 154 | 8.70 | 8.10 | 0.60 | 8.40 |
| 155 | 8.50 | 8.21 | 0.29 | 8.36 |
| 156 | 10.30 | 8.95 | 1.35 | 9.63 |
| 157 | 9.00 | 8.78 | 0.22 | 8.89 |
| 158 | 6.60 | 6.74 | -0.14 | 6.67 |
| 159 | 7.10 | 7.23 | -0.13 | 7.17 |
| 160 | 9.00 | 8.22 | 0.78 | 8.61 |
| 161 | 7.10 | 7.21 | -0.11 | 7.16 |
| 162 | 7.70 | 7.80 | -0.10 | 7.75 |
| 163 | 8.00 | 7.98 | 0.02 | 7.99 |
| 164 | 7.40 | 7.45 | -0.05 | 7.43 |
| 165 | 13.80 | 11.42 | 2.38 | 12.61 |
| 166 | 10.20 | 9.98 | 0.22 | 10.09 |
| 167 | 7.40 | 7.52 | -0.12 | 7.46 |
| 168 | 6.80 | 7.02 | -0.22 | 6.91 |
| 169 | 7.10 | 7.21 | -0.11 | 7.16 |
| 170 | 7.60 | 7.56 | 0.04 | 7.58 |
| 171 | 6.90 | 7.04 | -0.14 | 6.97 |
| 172 | 7.10 | 6.97 | 0.13 | 7.04 |
| 173 | 8.90 | 8.22 | 0.68 | 8.56 |
| 174 | 6.90 | 7.11 | -0.21 | 7.01 |
| 175 | 7.20 | 6.86 | 0.34 | 7.03 |
| 176 | 7.20 | 7.17 | 0.03 | 7.19 |
| 177 | 6.80 | 6.92 | -0.12 | 6.86 |
| 178 | 7.60 | 7.46 | 0.14 | 7.53 |
| 179 | 9.50 | 8.96 | 0.54 | 9.23 |
| 180 | 8.60 | 8.56 | 0.04 | 8.58 |
| 181 | 7.10 | 7.25 | -0.15 | 7.18 |
| 182 | 9.60 | 8.88 | 0.72 | 9.24 |
| 183 | 7.10 | 6.94 | 0.16 | 7.02 |
| 184 | 9.40 | 9.11 | 0.29 | 9.26 |
| 185 | 6.60 | 6.52 | 0.08 | 6.56 |
| 186 | 6.70 | 6.63 | 0.07 | 6.67 |
| 187 | 7.30 | 7.24 | 0.06 | 7.27 |
| 188 | 6.50 | 6.68 | -0.18 | 6.59 |
| 189 | 6.80 | 6.74 | 0.06 | 6.77 |
| 190 | 7.10 | 7.08 | 0.02 | 7.09 |
| 191 | 6.70 | 6.55 | 0.15 | 6.63 |
| 192 | 7.90 | 7.95 | -0.05 | 7.93 |
| 193 | 9.90 | 9.26 | 0.64 | 9.58 |
| 194 | 9.20 | 9.03 | 0.17 | 9.12 |
| 195 | 12.40 | 11.04 | 1.36 | 11.72 |
| 196 | 9.90 | 9.32 | 0.58 | 9.61 |
| 197 | 10.60 | 9.58 | 1.02 | 10.09 |
| 198 | 7.70 | 7.23 | 0.47 | 7.47 |
| 199 | 7.00 | 7.11 | -0.11 | 7.06 |
| 200 | 10.50 | 9.85 | 0.65 | 10.18 |
| 201 | 14.00 | 11.89 | 2.11 | 12.95 |
| 202 | 9.20 | 8.86 | 0.34 | 9.03 |
| 203 | 6.00 | 6.43 | -0.43 | 6.22 |
| 204 | 6.80 | 7.21 | -0.41 | 7.01 |
| 205 | 7.10 | 7.03 | 0.07 | 7.07 |
| 206 | 8.10 | 7.19 | 0.91 | 7.65 |
